# Cooperative Localization for Multi-AUVs Based on GM-PHD Filters and Information Entropy Theory

**DOI:** 10.3390/s17102286

**Published:** 2017-10-08

**Authors:** Lichuan Zhang, Tonghao Wang, Feihu Zhang, Demin Xu

**Affiliations:** School of Marine Science and Technology, Northwestern Polytechnical University, 127 West Youyi Road, Xi’an 710072, China; zlc@nwpu.edu.cn (L.Z.); xudm@nwpu.edu.cn (D.X.)

**Keywords:** cooperative localization (CL), multiple autonomous underwater vehicles (multi-AUVs), information entropy, probability hypothesis density (PHD) filter

## Abstract

Cooperative localization (CL) is considered a promising method for underwater localization with respect to multiple autonomous underwater vehicles (multi-AUVs). In this paper, we proposed a CL algorithm based on information entropy theory and the probability hypothesis density (PHD) filter, aiming to enhance the global localization accuracy of the follower. In the proposed framework, the follower carries lower cost navigation systems, whereas the leaders carry better ones. Meanwhile, the leaders acquire the followers’ observations, including both measurements and clutter. Then, the PHD filters are utilized on the leaders and the results are communicated to the followers. The followers then perform weighted summation based on all received messages and obtain a final positioning result. Based on the information entropy theory and the PHD filter, the follower is able to acquire a precise knowledge of its position.

## 1. Introduction

Localization is a primary issue for guaranteeing successful and efficient mission execution for autonomous underwater vehicles (AUVs) and other maritime robots. Common methods based on inertial measurement units (IMUs) have irreplaceable merit with respect to independencies, however, the accumulated error prevents high-accuracy localization in large-scale environments. In contrast to the use of more advanced IMUs, incorporating external information is a feasible solution. The authors of [1] considered the geomagnetic field sensor as an external information source during localization. Acoustic sensors such as sonars are also utilized in AUVs. Long baseline (LBL) [2,3] and ultra-short baseline (USBL) [4] systems often use sonars as an extension solution to support the localization. Sonar can also be used in underwater scenes of simultaneous localization and mapping (SLAM) [5]. The Doppler Velocity Log (DVL) is also a fundamental facility widely used for AUV navigation. An inertial navigation system (INV), DVL, and range measurement-based localization algorithm was designed for the AUV fleet [6]. Utilizing the DVL as an auxiliary sensor to INS, the authors of [7] exploited Unscented Kalman Filter (UKF) and Extended Kalman Filter (EKF) to locate the AUV positions . However, conventional methods work only in known areas (where essential facilities are deployed to offer standard reference), and are often restricted by the position of the sensors. Once the AUV flies beyond the border of these areas, the IMU system is the only option in the underwater environment. Cooperative localization (CL) is thus designed to address such problems by deploying sensors on the AUV. Moreover, CL can also extend the detection range to infinite regions, and enhance the reliability of the acoustic network.

According to the configuration of the navigation systems, CL is divided into parallel and the leader–follower structures. In the former structure, all AUVs carry same sensors, whereas in the latter, the follower carries lower accuracy navigation systems. Normally, systems in parallel structures are more expensive for implementation. Roumeliotis [8] performed some related work with Kalman filter-based multi robot cooperative localization strategies. The authors of [9] compared the algebraic and EKF methods of AUV cooperative localization. An extended information filter was employed to address CL issues in [10]. Uncertainty and communication constraints were also considered in [11,12,13]. The authors of [14] proposed an optimization approach to jointly localize the multi-AUVs. However, the clutter is often caused by the echo from the air–water surface, seabed or some other objects, and not considered in AUV CL applications. Traditional approaches often utilize the data association to address the clutter, which is invalid once the surveillance region is small. The nearest neighbor (NN) association is a basic approach. The authors of [15] investigated the application of the NN association in scenarios of abrupt motion tracking. Research on global NN and suboptimal NN was reported in [16], which provided approaches for multiple target tracking. The joint probabilistic data association (JPDA) method is also a solution to the data association issue. However, its accuracy decreases once the targets get to a small region [17]. Another widely utilized data association approach is the multiple-hypothesis tracking (MHT) method [18]. In [19], the MHT method was utilized to track the targets in cluttered images. Without data association steps, the probability hypothesis density (PHD) filter is a novel method to solve multiple target tracking problem in cluttered environment. Based on the PHD filter, it is possible to solve the disturbance caused by the clutter. In the PHD filter, all states and measures are modeled as set-based values in the format of a random finite set (RFS), which makes the PHD filter a promising approach to solving these issues. The PHD filter has been used in CL for vehicles [20,21] and the experiment implied its effectiveness in clutter environments. The PHD filter was also introduced to address the visual tracking issue [22,23], aiming to solve the number variation and noise corruption of the camera. In this paper, we apply the PHD filter to solve the AUV cooperative localization problem. Meanwhile, information entropy theory is a proper tool for determining the quality of the estimations. It has been used in optimization [24] and industrial areas to value how useful one message is [25,26,27]. Therefore, in this paper, underwater clutter is considered as a disturbance and the PHD filter incorporated with information entropy theory is simultaneously employed.

The remainder of this paper is organized as follows. Section 2 introduces the background of this paper and the mathematic models of the AUV and some sensors. Some basic knowledge about the information entropy theory is also included. In Section 3, the problem is stated and the detailed steps of the designed algorithm are provided. Section 4 provides the simulation results in two cases, both of which support the validity of the proposed method. Finally, Section 5 gives the conclusions and points out the prospects of this paper.

## 2. Background

### 2.1. Assumptions

**Figure 1:**
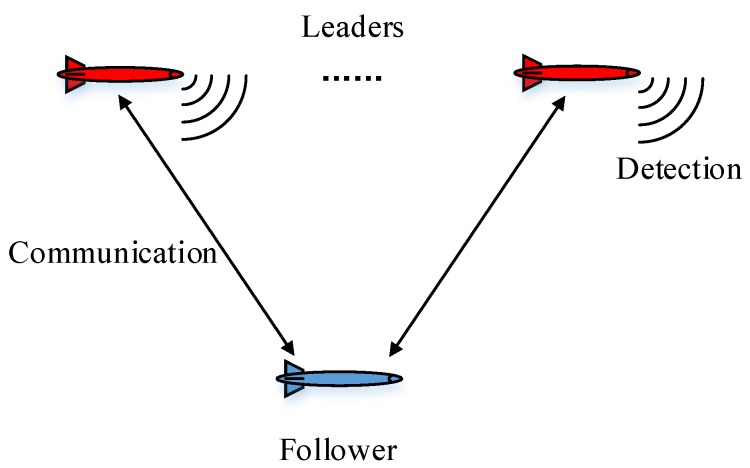
The leader–follower structure.

As shown in Figure 1:

1. The leader–follower structure is taken in order to achieve high accuracy with lower cost, in contrast to the parallel structure. Notice that the number of the leaders could be multiple, whereas the number of the followers is strictly limited to one.
2. Each AUV receives relative measurements of the others in the format of the range and bearing in local (body) coordinates. The global position of the follower can be calculated according to the measurement and the accurate position of the leader. However, the sonar cannot classify the clutter and the true measurements.
3. In this paper, only the communication between the leaders and the to-be-localized followers is considered. The measuring noise and connectivity uncertainty of the network are incorporated, whereas the time delay is beyond the scope of this paper.

### 2.2. Mathematic Models

#### 2.2.1. Model of AUV

**Figure 2:**
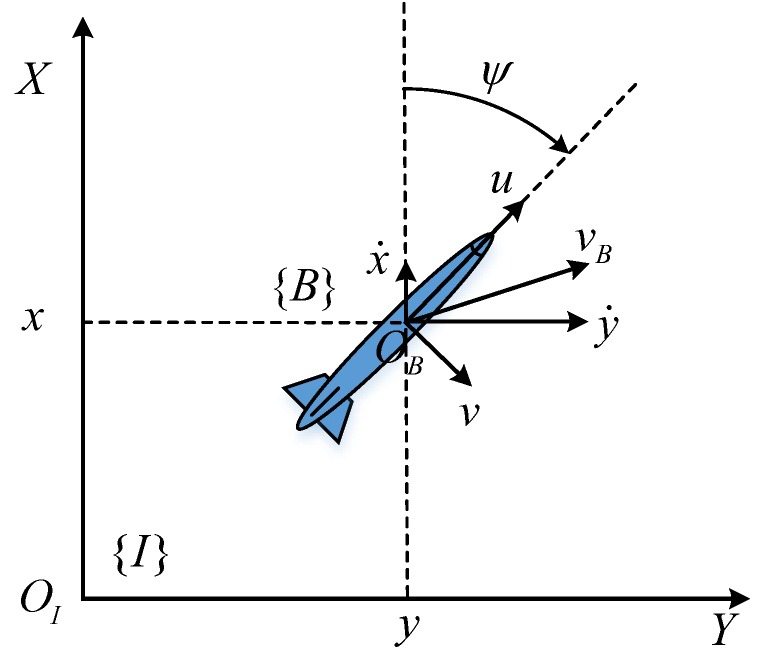
Two-dimensional model of the Autonomous Underwater Vehicle (AUV).

As shown in Figure 2, the AUV is modeled in the two-dimensional horizontal plane, neglecting the roll motion. According to [28,29], the kinematic model is utilized to describe the relationship between the AUV body-fixed reference frame ({B}-frame) and the Earth-fixed inertial reference frame ({I}-frame). Note that the {I}-frame is a simplified north—east—down (NED) system in the 2D horizontal plane, in which the *X* axis is pointing towards the north and the *Y* axis is pointing towards the east. The state vector of the AUV is then described as $X={\left[\begin{array}{cc} x & y \end{array}\right]}^{T}$, describing the position of the origin of the {B}-frame with respect to the {I}-frame (i.e., the position of the AUV in relation to {I}). ψ is the orientation of the {B}-frame with respect to {I}, i.e., the yaw of the AUV. $\nu ={\left[\begin{array}{cc} u & v \end{array}\right]}^{T}$ is the input of the AUV, denoting the linear velocity where *u* and *v* are the surge and sway speeds, respectively. *r* is the yaw rate of the AUV, i.e., $r=\dot{\psi }$.

Then the kinematic model of the AUV can be written as:

(1) $$\begin{array}{c} \dot{x}=u\cos\left(\psi \right)-v\sin\left(\psi \right)\\ \dot{y}=u\sin\left(\psi \right)+v\cos\left(\psi \right)\\ \dot{\psi }=r \end{array}$$

In the undersea environment, both the currents and the unexpected waves can interfere the movement of the AUV, and such noise is modeled as the additive systematic zero-mean white Gaussian noise vector $w{}_{X}\left(k\right)\in R^{2}$ at instant $t_{k}$. We have

(2) $$Q_{X}=E\left[w{}_{X}\left(k\right)w{}_{X}^{T}\left(k\right)\right]=\left[\begin{array}{cc} \sigma_{x}^{2} & \\ & \sigma_{y}^{2} \end{array}\right]$$

where $Q_{X}$ represents the covariance matrix of $w_{X}$.

Similarly, the noise of the yaw ψ is modeled as $w{}_{\psi }\left(k\right)\in R$, and the covariance is given by

(3) $$Q_{\psi }=E\left[w{}_{\psi }\left(k\right)w{}_{\psi }^{T}\left(k\right)\right]=\sigma_{\psi }^{2}$$

Thus, the real state (position) and yaw of the AUV can be written as:

(4) $$\begin{array}{c} X_{real}=X_{ideal}+w_{X}\\ \psi_{real}=\psi_{ideal}+w_{\psi } \end{array}$$

Only $X_{real}$ is used in the remainder of this paper, so it will be written as just *X* for clarity. Notice that since the leaders are assumed to carry high performance sensors, and the observations are carried out by the leaders, the yaw sensing error is neglected.

#### 2.2.2. Model of the Detecting Sonar

The AUV often relies on acoustic sonars to observe the surrounding environments. Notice that sonar signal processing includes transmitting, flying and receiving, etc.

##### A. Relative Distance-Sensing Part

The two-way time-of-flight sensor is utilized to compute the relative distance. Assuming that the time period between transmitting and receiving the signal is $t_{f}$ and the underwater sound velocity is *C*, the relative distance Dist can be expressed as

(5) $$Dist=t_{f}\cdot C$$

**Figure 3:**
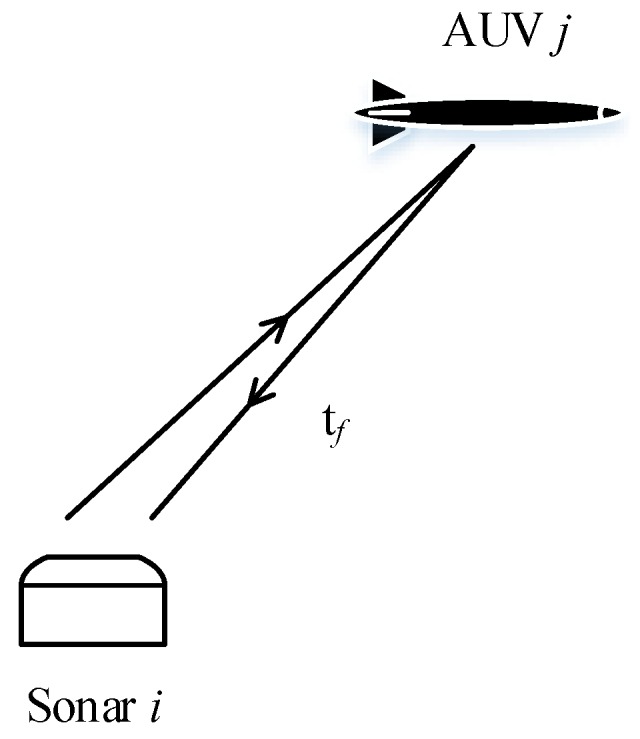
Schematic diagram of the relative distance detection.

Figure 3 shows that sonar *i* detects the AUV *j* at instant *k*. The distance measurement $z_{ij}^{d}\left(k\right)$ is then calculated by

(6) $$z_{ij}^{d}\left(k\right)=∥X_{j}-X_{i}∥+v_{p}\left(k\right)=\sqrt{{\left(x_{j}\left(k\right)-x_{i}\left(k\right)\right)}^{2}+{\left(y_{j}\left(k\right)-y_{i}\left(k\right)\right)}^{2}}+v_{p}\left(k\right)$$

where $X_{i}$ and $X_{j}$ are the states of the AUV *i* and the sonar *j*. $v_{p}\left(k\right)$ is the noise and is modeled by the additive stochastic zero-mean white Gaussian noise with variance $R_{p}\left(k\right)=\sigma_{p}^{2}$.

**Remark** **1.** Here we define the state of a sonar in the same way as the AUV for convenience. Normally, the sonar is fixed on the AUV in the head part sharing the forward direction with respect to the AUV. The third term of the vector $X_{j}$, denoting the yaw of the AUV, plays an important role in direction sensing.

##### B. Relative Direction-Sensing Part

**Figure 4:**
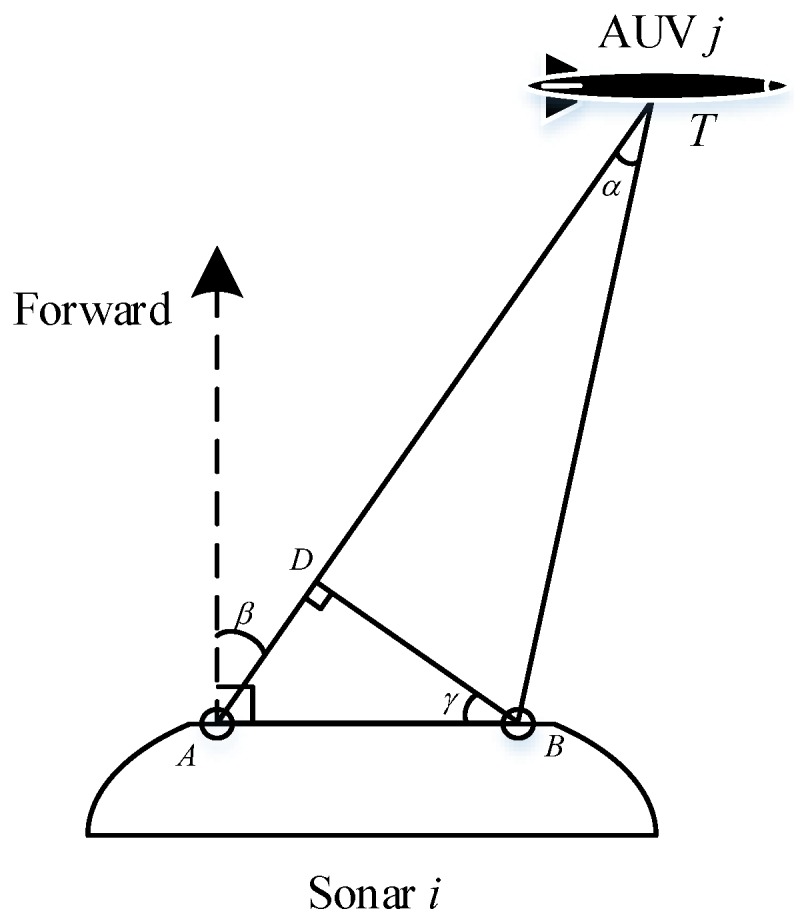
Schematic diagram of the relative direction detection.

The sonar is usually formed by a hydrophone array. Take Figure 4 as an example, A and B are two hydrophones in a sonar, and AUV *i* is the target to be detected. Signals from different direction reflect different hydrophones in a specific order. Hence, signal reflected by AUV *i* arrives at hydrophone B earlier than A.

Therefore, the time period $\tau_{AB}$ between the arrival instants can be used to compute the relative direction of the AUV against the sonar. Generally, the distance between the AUV and the detecting sonar (TA or TB) is much longer than AB. Assuming cosα=1, and AD=AB, we have

(7) $$AD=\tau_{AB}\cdot C$$

Thus, the relative direction is given by

(8) $$\beta =\arcsin\left(\tau_{AB}\cdot C/AB\right)+v_{\beta }\left(k\right)$$

where $v_{\beta }\left(k\right)$ is the stochastic zero-mean white Gaussian noise with variance $R_{d}\left(k\right)=\sigma_{d}^{2}$. $z_{ij}^{r}\left(k\right)$ denotes the relative direction measure between the sensor *j* and the target *j* at $t_{k}$.

**Figure 5:**
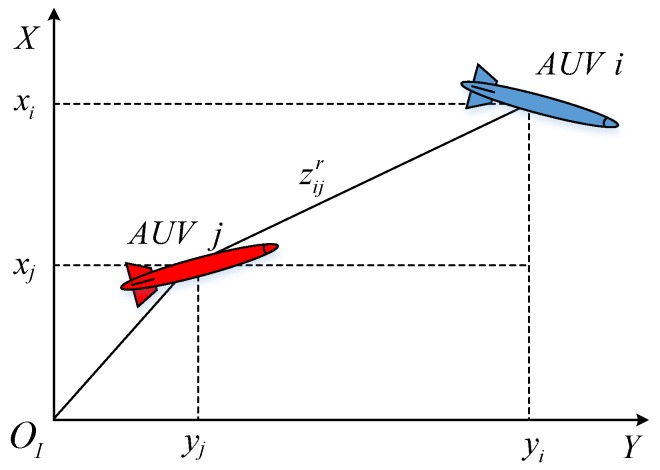
Position calculation.

Combing the relative distance and the direction (i.e. the relative measurements $z_{ij}^{r}$), the position of the AUV is acquired (see Figure 5).

### 2.3. PHD Filter

As aforementioned, observations from sonars also contain clutter due to the complicated underwater environment. Therefore, the standard Bayesian filter cannot obtain satisfactory estimations without data association. Compared to the standard Bayesian filters, finite set statistics analysis is a convenient approach for achieving the task without considering data association [30,31]. Hence, the RFS-based Gaussian mixture-probability hypothesis density (GM-PHD) filter is utilized.

Suppose that there are $N_{k-1}$ targets at instant $t_{k-1}$ with states

$X_{k-1}=\left\{x_{1}\left(k-1\right),\;x_{2}\left(k-1\right),\;\ldots ,\;x_{N_{k-1}}\left(k-1\right)\right\}$, and there are $N_{k}$ targets

$X_{k}=\left\{x_{1}\left(k\right),\;x_{2}\left(k\right),\;\ldots ,\;x_{N_{k}}\left(k\right)\right\}$ including newborn and dead ones. With the same manner, both $Z_{k-1}$ and $Z_{k}$ are defined. In an RFS manner, we have

(9) $$\begin{array}{c} X_{k}=\left\{x_{1}\left(k\right),\;x_{2}\left(k\right),\;\ldots ,\;x_{N_{k}}\left(k\right)\right\}\in F\left(X\right)\\ Z_{k}=\left\{z_{1}\left(k\right),\;z_{2}\left(k\right),\;\ldots ,\;z_{N_{k}}\left(k\right)\right\}\in F\left(Z\right) \end{array}$$

where F(X) and F(Z) denote the sets containing all states and observations, respectively. Notice that *Z* contains both the real measure of the target and the clutter.

Then, Equation (9) can be simplified as

(10) $$\begin{array}{c} X_{k}=\left(\underset{\zeta \in X(k-1)}{⋃}\;S_{k|k-1}\left(x\right)\right)\cup \Gamma_{k}\\ Z_{k}=\left(\underset{x\in X(k)}{⋃}\;\Theta_{k}\left(x\right)\right)\cup K_{k} \end{array}$$

where $S_{k|k-1}$ is the RFS of remaining targets at $t_{k-1}$, $\Gamma_{k}$ is the set of newborn ones at $t_{k}$, $\Theta_{k}\left(x\right)$ is the measure set of the targets, and $K_{k}$ is the clutters RFS.

Given the survival and detection rate as $p_{s,k}$ and $p_{d,k}$, the GM-PHD filter can be expressed in a Bayesian prediction-and-update form as

Prediction:

(11) $$\begin{array}{c} v_{k|k-1}\left(x\right)=v_{s,k|k-1}\left(x\right)+\gamma_{k}\left(x\right)\\ v_{s,k|k-1}\left(x\right)=p_{s,k}\sum_{j=1}^{J_{k-1}}\omega_{k-1}^{i}N\left(x;m_{s,k|k-1}^{j},P_{s,k|k-1}^{j}\right)\\ m_{s,k|k-1}^{j}=F_{k-1}m_{k-1}^{j}\\ P_{s,k|k-1}^{j}=Q_{k-1}+F_{k-1}P_{k-1}^{j}F_{k-1}^{T} \end{array}$$

Update:

(12) $$\begin{array}{c} v_{k}\left(x\right)=\left(1-p_{d,k}\right)v_{k|k-1}\left(x\right)+\sum_{z\in Z_{k}}v_{d,k}\left(x;z\right)\\ v_{k|k-1}\left(x\right)=\sum_{i=1}^{J_{k|k-1}}\omega_{k-1}^{i}N\left(x;m_{k|k-1}^{i},P_{k|k-1}^{i}\right)\\ v_{d,k}\left(x;z\right)=\sum_{j=1}^{J_{k|k-1}}\omega_{k-1}^{j}N\left(x;m_{k|k}^{j},P_{k|k}^{j}\right)\\ \omega_{k-1}^{i}=\frac{p_{d,k}\omega_{k-1}^{i}q_{k}^{i}\left(z\right)}{\kappa_{k}\left(z\right)+p_{d,k}\sum_{l=1}^{J_{k|k-1}}\omega_{k|k-1}^{l}q_{k}^{l}\left(z\right)}\\ q_{k}^{i}\left(z\right)=N\left(z;H_{k}m_{k|k}^{i},R_{k}+H_{k}P_{k|k-1}^{i}H_{k}^{T}\right)\\ m_{k|k}^{i}\left(z\right)=m_{k|k-1}^{i}+K_{k}^{i}\left(z-H_{k}m_{k|k-1}^{i}\right)\\ P_{k|k}^{i}=\left[I-K_{k}^{i}H_{k}\right]P_{k|k-1}^{i}\\ K_{k}^{i}=P_{k|k-1}^{i}H_{k}^{T}{\left(H_{k}P_{k|k-1}^{i}H_{k}^{T}+R_{k}\right)}^{-1} \end{array}$$

where $F_{k}$ and $H_{k}$ are the transition matrix and the measurement matrix, respectively. Note that if the transition function $f_{k}$ and the measurement function $h_{k}$ are nonlinearly presented, we can take their Jacobian matrices as $F_{k}$ and $H_{k}$. $\gamma_{k}$ denotes the Gaussian mixture and is given by

(13) $$\gamma_{k}\left(x\right)=\sum_{i=1}^{J_{\Gamma ,k}}\omega_{\Gamma ,k}^{i}N\left(x;m_{\Gamma ,k}^{i},P_{\Gamma ,k}^{i}\right)$$

Although the PHD filter estimates both the statements and its number, it does not have all of the track information. Thus, the identification of a specific state in its life cycle is not available. Hence, we have the information entropy theory to address this issue.

### 2.4. Information Entropy Theory

Normally, the standard PHD filter gives several results, which can fluctuate around the true states. However, in this paper, the follower needs only one estimation of its position. Thus, a proper method to calculate only one estimation with respect to all the results given by standard PHD filter is needed. Information entropy is one feasible tool to evaluate the quality or usefulness of the estimation. Therefore, it is employed to derive the weight coefficients for the PHD estimations.

Given a certain event $a_{i}$ with probability $p(a_{i})$, the amount of information brought by $a_{i}$ can be given by

(14) $$I\left(a_{i}\right)=\log_{b}\frac{1}{p(a_{i})}=-\log_{b}p\left(a_{i}\right)$$

where $I(a_{i})$ denotes the self-information and *b* is the base value of logarithm. Shannon defined the expected value of $I(a_{i})$ as information entropy, given by

(15) $$\begin{array}{cc} H(X) & =E\left[I(X)\right]=\sum_{i=1}^{n}p\left(x_{i}\right)I\left(x\right)\\ & =-\sum_{i=1}^{n}p\left(x_{i}\right)\log_{b}p\left(x_{i}\right) \end{array}$$

where *E* is the expect operator. Thus, information entropy can be a metric with respect to the uncertainty of an information source.

When considering several independent events (here we take two for example), the joint entropy and the conditional entropy can be written as

(16) $$H\left(X,Y\right)=-\sum_{i}\sum_{j}p\left(x_{i},y_{j}\right)\log p\left(x_{i},y_{j}\right)$$

(17) $$\begin{array}{cc} H(X|Y) & =\sum_{i}\sum_{j}p\left(x_{i},y_{j}\right)\log\frac{p(y_{j})}{p(x_{i},y_{j})}\\ & =-\sum_{i}\sum_{j}p\left(y_{j}\right)p\left(x_{i}|y_{j}\right)\log p\left(x_{i}|y_{j}\right) \end{array}$$

where *X* and *Y* denote two independent events with respect to $x_{i}$ and $y_{i}$, respectively.

Since H(X) and H(X|Y) describe the prior and posterior uncertainties, their difference is defined as mutual information as

(18) I(X;Y)=H(X)−H(X|Y)

When *X* is defined as n-dimensional Gaussian distribution, i.e., $X∼N(\mu_{Y},P_{Y})$, and taking *e* as the base to simplify the computation, the entropy can be written as

(19) $$H\left(X\right)=0.5\ln{\left(2\pi e\right)}^{n}\left|P_{X}\right|$$

## 3. Algorithm Design

In scenarios of *N* leader AUVs and one follower AUV *j*, all the AUVs can accurately obtain their positions on the sea surface. Once both the leaders and the follower dive into the water, the INS is utilized to localize them. The leaders can obtain accurate positions with the help of highly accurate navigation sensors, in contrast to the follower. To enhance the positioning accuracy of the follower, the leaders should also estimate the position of the follower via relative measures. However, due to the complicated underwater environment, the clutter issue is quite challenging. The GM-PHD filter is thus utilized to filter the clutter from the whole measurement set.

However, all the leaders give different estimations with respect to the same follower. For better execution, the follower needs only one accurate position of itself. In this paper, a specific algorithm is designed for the follower to cope with all the estimations from different sources properly. The process of the algorithm is as follows.

**Step 1:** Measuring. At $t_{k}$, the leaders measure the follower. For instance, the leader AUV *i* collects the observations $z_{k-1}^{ij}$ including the true measurements of the follower AUV *j*${\hat{z}}_{k-1}^{ij}$ and the clutter $c_{k}^{ij}$, i.e., $z_{k-1}^{ij}=\left\{{\hat{z}}_{k-1}^{ij}\right\}\cup \left\{c_{k-1}^{ij}\right\}$.

**Step 2:** Calculating. With the help of the GM-PHD filter, each leader computes the estimated positions of the follower in the format of ${\hat{X}}_{k|k}^{ij,t}$. Moreover, according to Equations (17) and (19), the entropy of ${\hat{X}}_{k|k-1}^{ij,t}$ and ${\hat{X}}_{k|k}^{ij,t}$ are also calculated by the leaders, denoted as

(20) $$\begin{array}{c} H\left({\hat{X}}_{k|k-1}^{ij,t}\right)=0.5\ln{\left(2\pi e\right)}^{n}\left|P_{k|k-1}\right|\\ H\left({\hat{X}}_{k|k}^{ij,t}\right)=0.5\ln{\left(2\pi e\right)}^{n}\left|P_{k|k}\right| \end{array}$$

where $P_{k|k-1}$ and $P_{k|k}$ are the prior and posterior covariance matrices, respectively. Thus, the mutual information is given by

(21) $$I_{k}^{ij,t}=I\left({\hat{X}}_{k|k-1}^{ij,t};{\hat{X}}_{k|k}^{ij,t}\right)=H\left({\hat{X}}_{k|k-1}^{ij,t}\right)-H\left({\hat{X}}_{k|k}^{ij,t}\right)$$

which describes the amount of uncertainty eliminated by ${\hat{X}}_{k|k}^{ij,t}$.

**Step 3:** Communicating. Leaders broadcast both the estimated positions and the corresponding mutual information values.

**Step 4:** Fusing. The follower AUV *j* collects all the estimations and mutual information values from the leaders. Note that the number of the data pairs is usually larger than the number of the leaders due to the property of the PHD filter. The more uncertainty is eliminated, the better the estimation. Assuming that the leader AUV *i* gives $M_{i}$ pairs of the results, the weighted summation of all the estimations is given by

(22) $${\hat{X}}_{k|k}^{j}=\sum_{i=1}^{N}\sum_{t=1}^{M_{i}}w_{it}{\hat{X}}_{k|k}^{ij,t}$$

where $w_{it}$ is the normalized weighting coefficient computed by

(23) $$w_{it}=\frac{I({\hat{X}}_{k|k-1}^{ij,t};{\hat{X}}_{k|k}^{ij,t})}{\sum_{i=1}^{N}\sum_{t=1}^{M}I({\hat{X}}_{k|k-1}^{ij,t};{\hat{X}}_{k|k}^{ij,t})}$$

Thus, the calculated ${\hat{X}}_{k|k}^{j}$ is the final estimation of the follower AUV *j*, which is then broadcasted by the follower to the leaders, for further PHD filtering steps.

**Step 5:** Back to Step 1.

**Figure 6:**
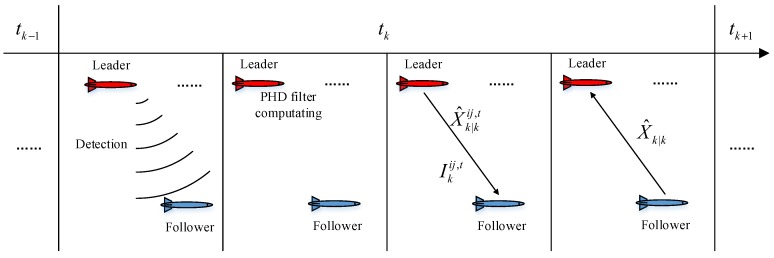
Working procedure of the proposed algorithm. PHD: Probability Hypothesis Density.

Figure 6 presents a brief procedure of the proposed algorithm.

## 4. Simulation and Results

Simulations are designed to validate the proposed method, under the assumed communication conditions with noise and intermittent network failure. Two scenarios are designed as follows.

### *Scenario 1:* 

There were two leading AUVs in this scenario, AUV 1 and AUV 2, and they both took straight flights with a speed of 2 kn. Starting from the origin of the Earth-fixed frame, their directions were northeast (45${}^{\circ }$ to the north) and west, respectively. Their measuring frequency was set to 1 Hz. The follower AUV 3 took a circular motion on the 2D square surveillance region with the *x*-range [−200 m, 300 m] and *y*-range [−400 m, 200 m]. AUV 3 started its flight at (0, 0) with cruising speed of 5 kn and yaw angular velocity 0.05 rad/s. Note that the angular velocity is positive clockwise in the NED frame. The motion noise was assumed to be zero mean white Gaussian noise with covariance matrix $Q=\mathrm{diag}(\left[1\;m^{2},\;1\;m^{2},\;10^{-4}{\mathrm{rad}}^{2}\right])$, while the covariance matrix of the measurement noise was $R=\mathrm{diag}(\left[25\;m^{2},\;25\;m^{2}\right])$. The clutter was uniformly distributed over the surveillance region and the number of the clutter observations followed $N_{Clutters}∼P\left(\lambda_{Clutters}\right)$, where $\lambda_{Clutters}$ is calculated by the area of the region and the occurrence probability $p_{Clutters}$. Here the $p_{Clutters}=12.5\times 10^{-6}/m^{2}$. The network failure ratio was set to 10% and the detection failure ratio was set to 2%.

**Figure 7:**
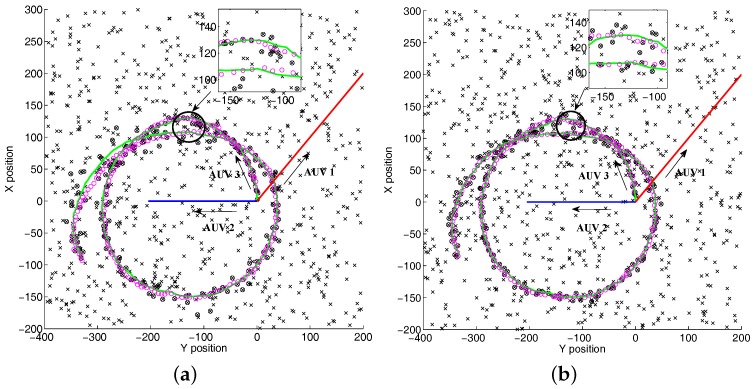
Trajectories, clutter, and measurements in Scenario 1. (**a**) Result with respect to AUV 1 only; (**b**) Result with respect to AUV 2 only.

The trajectories under this condition are shown in Figure 7.

The red, blue and green lines in Figure 7a,b are the actual trajectories of the leaders AUV 1 and 2, and the follower AUV 3, respectively. Black circles represent the real measures of AUV 3 and the "×" markers show the clutter. The magenta circles give the results of the standard PHD filter executed by one single leader.

**Figure 8:**
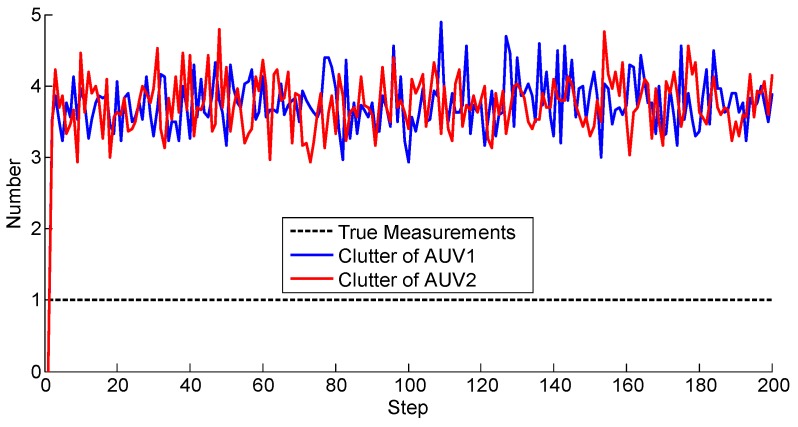
Number of the clutter observations.

As aforementioned, the number of the clutter observations is randomly generated according to the Poisson distribution. In this simulation, the number is plotted in Figure 8.

**Figure 9:**
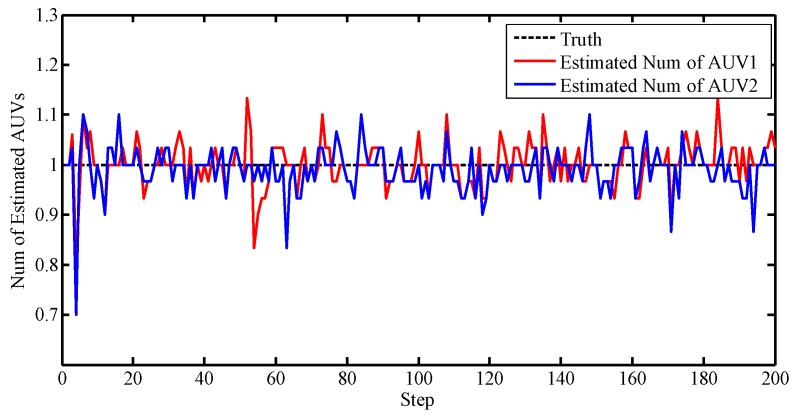
Number of estimations.

We note that the true measures are merged in the clutter. Thus, the PHD filter was incorporated to eliminate the effects of the clutter during filtering. Figure 9 illustrates the number of the PHD estimations.

Notice that without data association, the estimated number fluctuates around the true value. From Figure 9, it is observed that the PHD cannot eliminate the clutter completely, but compared with Figure 8, the number has been adjusted to a small range.

**Figure 10:**
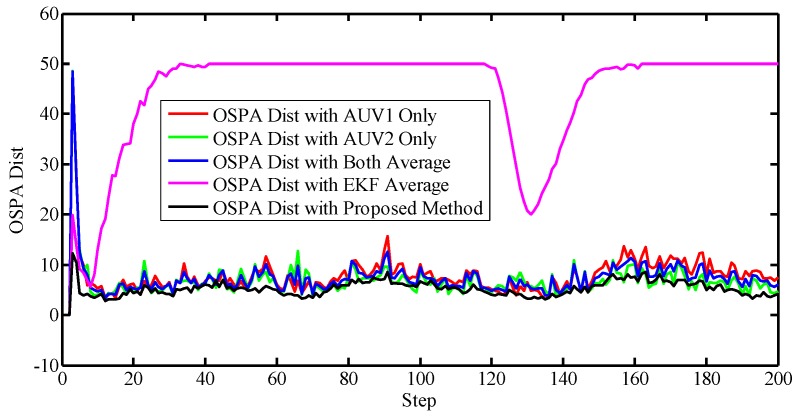
Positioning error. OSPA: Optimal Sub-Pattern Assignment, Dist: Distance, EKF: Extended Kalman Filter.

To better evaluate the proposed approach, a metric was needed to justify the accuracies. Notice that the standard Root-Mean-Square Error (RMSE) cannot be directly used due to the random number of the set-valued states. Therefore, the Optimal Sub-Pattern Assignment (OSPA) was utilized to describe the accuracy of the localization. OSPA was first proposed in [32] as a metric in the multi-object filtering field, describing the miss-distance, or error, between the reference values and the estimated ones. Note that using OSPA, the number of reference values and the estimations do not have to be the same. In other words, OSPA offers a metric between two sets regardless the cardinalities. A larger value, i.e., larger miss-distance, indicates worse accuracy. Here we plotted the OSPA distance lines of different algorithms in Figure 10. Note that the OSPA error lines were obtained after 100 Monte Carlo simulations, and the saturation value of the OSPA is set to 50.

The EKF with the nearest neighbor rule was also carried out as the comparison to the PHD filter. Generally, the EKF performs the poorest, implying the superiority of the PHD filter. Also, the OSPA distance of the proposed method is smalles than both the methods exploiting one AUV and the alternative solutions using them both, which illustrates the advantage of the proposed method.

### *Scenario 2:* 

**Figure 11:**
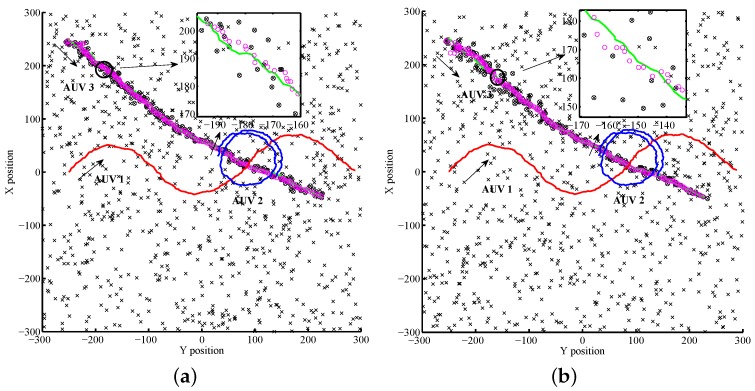
Trajectories, clutter and measurements in Scenario 2. (**a**) Result with respect to AUV 1 only; (**b**) Result with respect to AUV 2 only.

In this scenario, the AUVs cruise in different manner. The cruise velocities were 5 kn for the leaders AUV 1 and AUV 2, and 2 kn for the follower AUV 3. As shown in Figure 11, the AUV 1 started from (0, −250) in the west of the origin of the NED frame, to the east in a sine wave routine, while the AUV 2 took a circular path from (30, 30), with yaw rate 0.05 rad/s. AUV 3 traveled on the horizontal region with both the *x*-range and *y*-range [−300,300], from the northwest (−250, 250) straight to the southeast in 3 kn. Compared to Scenario 1, the network failure rate increased to 20% and the motion (systematic) noise of the leaders was also considered. The rest parameters were set up in the same manner as for Scenario 1.

The trajectories are described as red line (leader AUV 1), blue line (leader AUV 2), and green line (follower AUV 3) in Figure 11a,b. The real measures of AUV 3 are denoted as black circles and the "×" markers represent the clutter. The results of the standard PHD filter executed by one single leader are given by the magenta.

**Figure 12:**
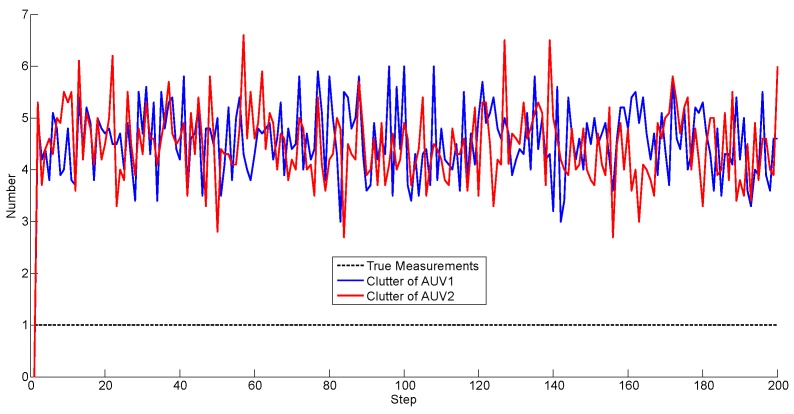
Number of the clutter observations.

**Figure 13:**
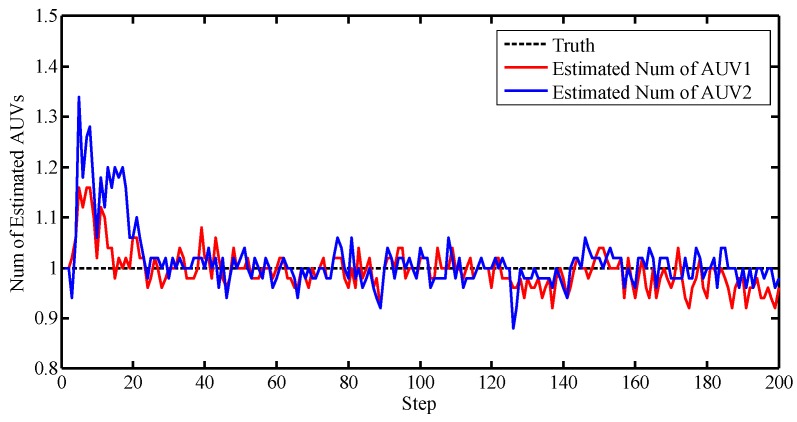
Number of estimations.

The number of the clutter observations and PHD estimations are given in Figure 12 and Figure 13.

**Figure 14:**
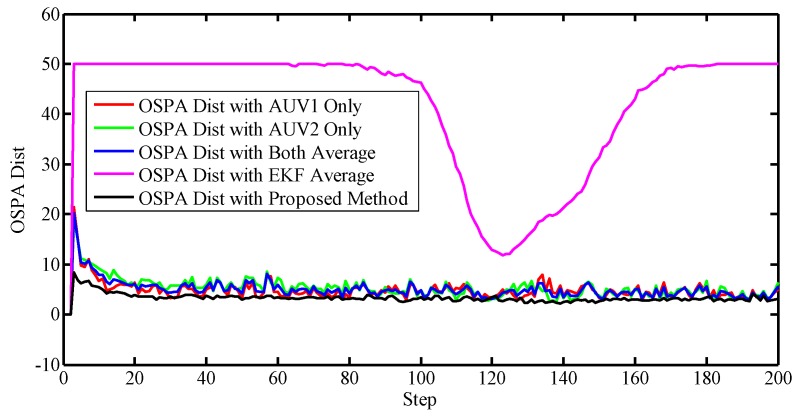
Positioning error.

Figure 12 and Figure 13 illustrate that the PHD filter still managed to reduce the influence of the clutter. The OSPA distances with respect to different algorithms are shown in Figure 14.

In this scenario, the proposed method still achieved the best performance compared to the EKF, the single PHD filter, and the average of them.

However, the computational load slightly increases due to the calculation of mutual information. Running on a computer with Intel(R) Core(TM) i7-4710MQ CPU at 2.5GHz, the computing time of 50 executions of the standard PHD filter and the proposed method in different scenarios are given in the Table 1.

According to the simulation results, the PHD filter and information entropy-based cooperative localization algorithm achieved higher accuracy and stability compared to the state-of-the-art approaches. The computation burden is increased by computing the mutual information, but according to the Table 1, the loads were only increased by around 8%.

## 5. Conclusions

In this paper, an information entropy-based GM-PHD filter was employed to tackle the cooperative localization issue. By exploiting both the GM-PHD filter and the information entropy theory, the proposed method can obtain more accurate results in contrast to the standard GM-PHD filter and the EKF approach with NN association strategy. Mathematical models were derived and a simulation was designed, which implied that the designed method performed well in scenarios of clutter, intermittent connection, and detection failure.

There are also some prospects that need to be investigated in future. One is to explore the performance of the proposed method in the communication environment with time delay. The time delay issue results in a bottleneck of underwater CL, which can decrease the positioning accuracy. A deeper insight into this is required. Also, observability is an important feature of the multi-AUV system [33]. The influence of the observability in the proposed method should be investigated. Moreover, how to utilize the proposed method in scenarios of multiple followers is the key to applying this algorithm more widely.

Besides the theoretical improvements of the proposed algorithm, experimental validations should also be executed in the future for a more convincing conclusion. Primary experiments can be implemented in artificial environments like swimming pools with preset clutter, or lakes, and the AUVs can be replaced by sonar and acoustic communication units for simplification. Then, several AUVs or some other underwater robots with similar motion patterns modeled in this paper should be exploited and a sea experiment will be needed to validate the proposed method in practice.

## Figures and Tables

**Table 1:** Computing time of the standard PHD filter and the proposed method.

	Standard PHD Filter (s)	Proposed Method (s)	Increased Percentage (%)
**Scenario 1**	211.602	229.887	8.64
**Scenario 2**	247.366	267.481	8.13

## References

[1] Huang Y., Hao Y. Method of separating dipole magnetic anomaly from geomagnetic field and application in underwater vehicle localization. Proceedings of the 2010 IEEE International Conference on Information and Automation.

[2] Matos A., Cruz N., Martins A., Lobo Pereira F. Development and implementation of a low-cost LBL navigation system for an AUV. Proceedings of the OCEANS ’99 MTS/IEEE.

[3] Paull L., Saeedi S., Seto M., Li H. (2014). AUV Navigation and Localization: A Review. IEEE J. Ocean. Eng..

[4] Allotta B., Bartolini F., Caiti A., Costanzi R., Di Corato F., Fenucci D., Gelli J., Guerrini P., Monni N., Munafò A. (2015). Typhoon at CommsNet13: Experimental experience on AUV navigation and localization. Annu. Rev. Control.

[5] Stachniss C., Leonard J.J., Thrun S., Siciliano B., Khatib O. (2016). Simultaneous Localization and Mapping. Springer Handbook of Robotics.

[6] Allotta B., Pugi L., Costanzi R., Vettori G. Localization algorithm for a fleet of three AUVs by INS, DVL and range measurements. Proceedings of the 2011 15th International Conference on Advanced Robotics (ICAR).

[7] Karimi M., Bozorg M., Khayatian A. A comparison of DVL/INS fusion by UKF and EKF to localize an autonomous underwater vehicle. Proceedings of the 2013 First RSI/ISM International Conference on Robotics and Mechatronics (ICRoM).

[8] Huang G.P., Trawny N., Mourikis A.I., Roumeliotis S.I. (2011). Observability-based consistent EKF estimators for multi-robot cooperative localization. Auton. Robots.

[9] Zhang L., Wang J., Wang T., Liu M., Gao J. Optimal formation of multiple AUVs cooperative localization based on virtual structure. Proceedings of the OCEANS 2016 MTS/IEEE Monterey.

[10] Rui G., Chitre M. Cooperative multi-AUV localization using distributed extended information filter. Proceedings of the 2016 IEEE/OES Autonomous Underwater Vehicles (AUV).

[11] Nerurkar E.D., Roumeliotis S.I. Asynchronous Multi-Centralized Cooperative Localization. Proceedings of the 2010 IEEE/RSJ International Conference on Intelligent Robots and Systems (IROS).

[12] Nerurkar E.D., Roumeliotis S.I. A communication-bandwidth-aware hybrid estimation framework for multi-robot cooperative localization. Proceedings of the 2013 IEEE/RSJ International Conference on Intelligent Robots and Systems (IROS).

[13] Nerurkar E.D., Zhou K.X., Roumeliotis S.I. A hybrid estimation framework for cooperative localization under communication constraints. Proceedings of the 2011 IEEE/RSJ International Conference on Intelligent Robots and Systems (IROS).

[14] Ferreira B.Q., Gomes J., Soares C., Costeira J.P. Collaborative localization of vehicle formations based on ranges and bearings. Proceedings of the 2016 IEEE Third Underwater Communications and Networking Conference (UComms).

[15] Zhou T., Lu Y., Lv F., Di H., Zhao Q., Zhang J. (2015). Abrupt motion tracking via nearest neighbor field driven stochastic sampling. Neurocomputing.

[16] Konstantinova P., Udvarev A., Semerdjiev T. A study of a target tracking algorithm using global nearest neighbor approach. Proceedings of the International Conference on Computer Systems and Technologies (CompSysTech ’03).

[17] Fitzgerald R. (1985). Track biases and coalescence with probabilistic data association. IEEE Trans. Aerosp. Electr. Syst..

[18] Blackman S.S. (2004). Multiple hypothesis tracking for multiple target tracking. IEEE Aerosp. Electr. Syst. Mag..

[19] Chenouard N., Bloch I., Olivo-Marin J.C. (2013). Multiple hypothesis tracking for cluttered biological image sequences. IEEE Trans. Pattern Anal. Mach. Intell..

[20] Zhang F., Buckl C., Knoll A. (2014). Multiple Vehicle Cooperative Localization with Spatial Registration Based on a Probability Hypothesis Density Filter. Sensors.

[21] Goli A.S., Far H.B., Fapojuwo O.A. Cooperative Multi-sensor Multi-vehicle Localization in Vehicular Adhoc Networks. Proceedings of the 2015 IEEE International Conference on Information Reuse and Integration.

[22] Feng P., Wang W., Dlay S., Naqvi S.M., Chambers J. (2017). Social Force Model-Based MCMC-OCSVM Particle PHD Filter for Multiple Human Tracking. IEEE Trans. Multimedia.

[23] Zhou X., Li Y., He B., Bai T. (2014). GM-PHD-Based Multi-Target Visual Tracking Using Entropy Distribution and Game Theory. IEEE Trans. Ind. Inform..

[24] Zhou X., Zhang Y., Hao S., Li S. A new approach for noise data detection based on cluster and information entropy. Proceedings of the 2015 IEEE International Conference on Cyber Technology in Automation, Control, and Intelligent Systems (CYBER).

[25] Zou G., Ma L., Zhang L., Mo L. An indoor positioning algorithm using joint information entropy based on WLAN fingerprint. Proceedings of the 2014 International Conference on Computing, Communication and Networking Technologies (ICCCNT).

[26] Mocnej J., Lojka T., Zolotov T. Using information entropy in smart sensors for decentralized data acquisition architecture. Proceedings of the 2016 IEEE 14th International Symposium on Applied Machine Intelligence and Informatics (SAMI).

[27] Guo X., Liu X., Chen G., Chang L., Li X. Missile weapon system-of-systems optimization method based on information entropy. Proceedings of the 2016 International Conference on Computer, Information and Telecommunication Systems (CITS).

[28] Fossen T.I. (1994). Guidance and Control of Ocean Vehicles.

[29] Lapierre L., Jouvencel B. (2008). Robust nonlinear path-following control of an AUV. IEEE J. Ocean. Eng..

[30] Mahler R.P.S. (2004). “Statistics 101” for multisensor, multitarget data fusion. IEEE Aerosp. Electr. Syst. Mag..

[31] Mahler R. (2013). “Statistics 102” for Multisource-Multitarget Detection and Tracking. IEEE J. Sel. Top. Signal Process..

[32] Schuhmacher D., Vo B.T., Vo B.N. (2008). A Consistent Metric for Performance Evaluation of Multi-Object Filters. IEEE Trans. Signal Process..

[33] Arrichiello F., Antonelli G., Aguiar A.P., Pascoal A. Observability metric for the relative localization of AUVs based on range and depth measurements: Theory and experiments. Proceeding of the 2011 IEEE/RSJ International Conference on Intelligent Robots and Systems (IROS).

